# Congenital Rubella Syndrome as a possible cause for persistent thrombocytopenia in early infancy: The Forgotten Culprit

**DOI:** 10.4322/acr.2021.386

**Published:** 2022-06-06

**Authors:** Jogender Kumar, Venkataseshan Sundaram, Kirti Gupta, Anmol Bhatia, Gurwinder Kaur, Sourabh Dutta

**Affiliations:** 1 Post Graduate Institute of Medical Education & Research (PGIMER), Department of Pediatrics, Chandigarh, India; 2 Post Graduate Institute of Medical Education & Research (PGIMER), Department of Histopathology, Chandigarh, India; 3 Post Graduate Institute of Medical Education & Research (PGIMER), Department of Radiodiagnosis, Chandigarh, India

**Keywords:** Rubella Syndrome, Congenital, Thrombocytopenia, Pathology, Neuropathology, Autopsy

## Abstract

We present a case of a late preterm intrauterine growth-restricted neonate with isolated and persistent severe thrombocytopenia. At birth, the neonate did not have a complete clinical spectrum of congenital rubella syndrome (CRS) but later developed peculiar findings that helped clinch the diagnosis. The neonate also had interstitial pneumonia and died secondary to superimposed acute viral infection leading to acute respiratory distress syndrome. The serology was positive for IgM antibodies against the rubella virus. The constellation of clinical manifestations of congenital rubella in the presence of positive IgM antibody against rubella and consistent histopathology confirmed the diagnosis of CRS.

## INTRODUCTION

Thrombocytopenia (platelet count less than 150,000/µL) is common in sick neonates admitted to neonatal intensive care units and is mainly attributed to neonatal sepsis. However, persistent severe thrombocytopenia (platelet count less than 50,000/µL) in a non-septic neonate is rare and warrants thorough evaluation. Congenital rubella syndrome (CRS) is one of the uncommon causes of persistent thrombocytopenia in infancy. In the first trimester of pregnancy, rubella infection in an unimmunized mother may lead to severe consequences, including miscarriages, stillbirths, and a constellation of severe birth defects in the neonate. Therefore, in countries where vaccination coverage against rubella is poor, it should be considered one of the differential diagnoses of persistent severe thrombocytopenia. Herein, we present a case of persistent thrombocytopenia, which turned out to be congenital rubella syndrome (CRS).

## CASE REPORT

A baby girl was born at 35 weeks, weighing 938 g, by cesarean section because of meconium-stained liquor and fetal distress. The antenatal period was uncomplicated except for maternal pregnancy-induced hypertension (PIH) and fetal growth restriction. Since birth, the neonate developed respiratory distress and was referred to the neonatal intensive care unit (ICU). At 68 hours of life, she had one episode of epistaxis. Her heart rate was 142/minute, respiratory rate of 69/minute with mild subcostal retractions, normal saturation, and capillary refill time. She weighed 938 g (<3^rd^ centile), had an occipital-frontal circumference of 27.5cm (<3^rd^ centile), and a ponderal index of 1.58 (<3^rd^ centile). Systemic examination was normal, and there was no evidence of major congenital malformation. Investigations revealed normal hemoglobin-15.6 gm/dL (range 14-22 gm/dL), white blood cell count-11900/µL (range 10000- 26000/µL), but low platelet count- 4000/µL (range 100,000-450,000/µL) (Table 1), elevated prothrombin time- 22s (range 11.6- 16.2s) and activated partial thromboplastin time - 59s (range 34.3- 44.8s). She received two units of platelets and one unit of fresh frozen plasma. Renal ultrasonography showed a small right kidney (1.8 X 2.3 cm) with hydronephrosis, suggesting cystic dysplasia of the right kidney. The skeletal survey did not show metaphysitis or radioulnar synostosis.

**Table 1:** Diagnostic investigation of the index case

Workup	Result
Microbiology	**Blood culture** (Day 4, 10, 17 and Postmortem): sterile. **Urine for fungal smear** (Day 10 and 17): Negative. **Cerebrospinal fluid** (Day 4): Sugar- 41 mg/dl, Protein- 168 mg/dl, Cells- 120, all polymorphs. Gram-stain and culture - sterile. **TORCH profile (Baby)**: Toxoplasmosis ELISA- IgG and IgM negative; Rubella chemiluminescence- IgM reactive; CMV chemiluminescence- nonreactive; Herpes Simplex ELISA- negative. **TORCH profile (Mother)**: Toxoplasmosis ELISA- IgG and IgM negative; Rubella chemiluminescence- IgM nonreactive, IgG - reactive; CMV chemiluminescence- nonreactive; Herpes Simplex ELISA- negative
Immunology	**Immunoglobulin profile**: IgM < 11 mg/dL (40-160 mg/dL); IgG 786 mg/dL (300-900 mg/dL); IgA 61 mg/dL (15-70 mg/dL) HIV ELISA – Nonreactive **Mother’s antinuclear antibody and antineutrophil cytoplasmic antibodies** - Negative
Radiological	**Chest X-ray** (Day 1): Hyper inflated lungs with heterogeneous ill-defined infiltrates? Meconium aspiration syndrome. **Chest X-ray** (Day 17): Diffuse infiltrative opacification in the peripheral lung field? Interstitial pneumonitis Echocardiography: 3mm patent ductus arteriosus with small atrial septum defect. **X-ray of Long bones**- normal, no metaphysitis or radioulnar synostosis. **Ultrasound of abdomen and pelvis**: Cystic dysplasia of right kidney with loss of corticomedullary differentiation, and mild hydroureteronephrosis of the right kidney. **Ultrasound cranium**: Few echogenic foci in bilateral basal ganglia? Calcification
Eye evaluation	(day 5)- No cataract or chorioretinitis. Day 21- Sutural cataract and Fundus couldn’t be visualized
Platelet counts	4000/µL on day 3, 16000/µL on day 5, 21000/µL on day 7, 60000/µL on day 14, 46000/µL on day 21, and 34000/µL on day 29 of life.

The possibility of sepsis with disseminated intravascular coagulation (DIC) was considered, and intravenous antibiotics were started. Lumbar puncture was suggestive of meningitis (Sugar- 41 mg/dL, Protein- 168 mg/dL, Cells- 120, all polymorphs; Culture- sterile), for which meropenem was given for 21 days. She showed improvement in clinical as well as laboratory parameters except for thrombocytopenia. Given the persistent thrombocytopenia, the possibility of neonatal alloimmune thrombocytopenia (NAIT) was considered, and platelet transfusions along with two doses (1 g/kg) of intravenous immunoglobulin (IVIG) were given over 48 hours. However, there was no sustained improvement in the platelet count. The mutation analysis for Human platelet antigen (HPA) could not be done. The echocardiography depicted a persistent small hemodynamically insignificant patent ductus arteriosus. The initial fundus evaluation did not show any evidence of chorioretinitis. During the hospital stay, she was stable on full enteral feeding by orogastric tube and nasal prongs oxygen and gained weight. On day 21 of life, a sutural cataract was detected while the fundus examination was attempted, following which suspicion of TORCH infections was initiated.

The initial cranial ultrasound on day 4 of life was normal. A repeated examination on day 22 showed few echogenic foci (suspected calcification) in the bilateral basal ganglia. A TORCH serology sent at that juncture revealed rubella-specific IgM antibody positive. Since she had stigmata of congenital rubella syndrome (i.e., growth restriction, microcephaly, cataract, thrombocytopenia) and the serology was positive, she was diagnosed as a confirmed case of congenital rubella syndrome (CRS). The baby was shifted to step-down care and was managed with nasal prongs, oxygen, and tube feeds. The chest radiograph done at that time was suggestive of interstitial pneumonia. One day before her demise, the mother had an upper respiratory tract infection, and the baby also worsened simultaneously. As the infant-mother stayed together, the possibility of viral pneumonia was kept, and supportive management (oxygen, fluids, antibiotics) was given. Over the next 18 hours, she developed progressive hypoxemia and respiratory distress syndrome (ARDS). She was mechanically ventilated, but she continued to worsen and succumbed to her illness. The blood culture sent at the time of worsening was sterile, and serum procalcitonin was also negative (0.3 ng/mL). Table 1 summarizes the laboratory work up.

To summarize, this was a preterm IUGR neonate with cataract, persistent ductus arteriosus, microcephaly, thrombocytopenia, and pneumonia. A clinical diagnosis of congenital rubella syndrome (CRS) with interstitial pneumonia superimposed with acute respiratory distress syndrome was considered.

## AUTOPSY FINDINGS

**Figure 1:**
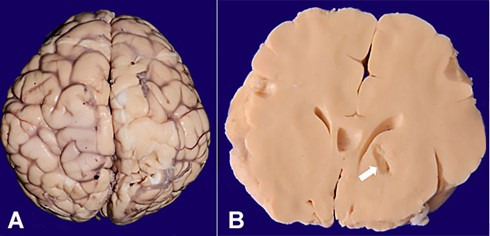
Gross view of the brain **A** – Cerebral hemispheres viewed from superior aspect with normal gyral pattern; **B** – Coronal slice depicting cystic lesion consistent with infarct in basal ganglia (white arrow)

A complete autopsy was performed after informed consent. There was microcephaly with a brain weight of 300 g (normal weight for this age 430 gm); however, the gyri and sulci were normal, and the gyral pattern was normal (Figure 1A). The meninges were unremarkable. No uncal or tonsillar herniation was noted. On coronal slicing, the right basal ganglia revealed areas of softening and cystic infarct (1cm in size) (Figure 1B).

**Figure 2:**
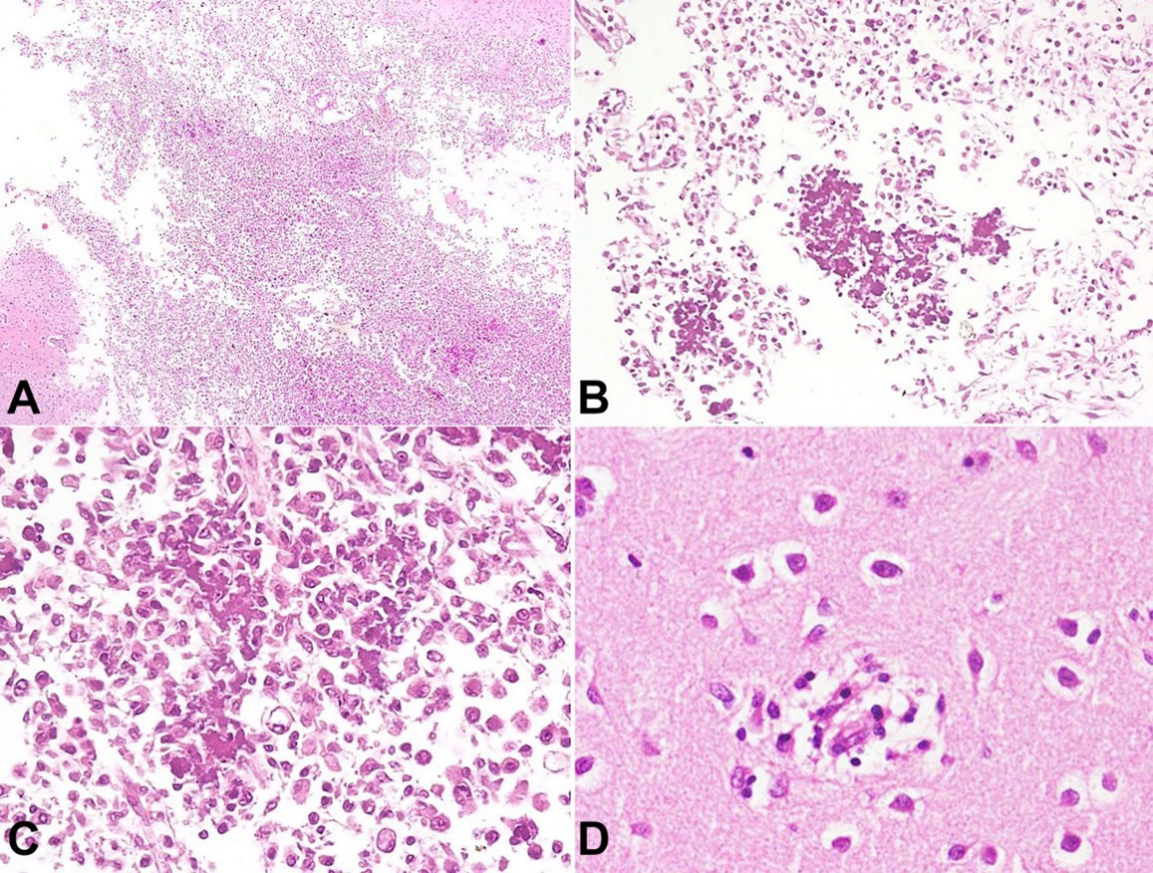
Photomicrograph of basal ganglia **A** – Low magnification demonstrates the cystic infarct (H&E x100); **B** – Areas of calcification noted within the infarcted region. Macrophages are seen in the background (H&E x200); **C** – High magnification of areas of calcification (H&E x400); **D** – Microglial nodule (H&E x400)

There was squaring of the angle of lateral ventricles. The brain stem, including the midbrain, pons, and medulla, was unremarkable. On microscopy, both basal ganglia showed areas of cystic infarct with plenty of macrophages. The background showed reactive astrocytes and dystrophic calcification within these infarction areas (Figures 22B, and 2C). Few microglial nodules were noted at the periphery of the infarct (Figure 2D).

**Figure 3:**
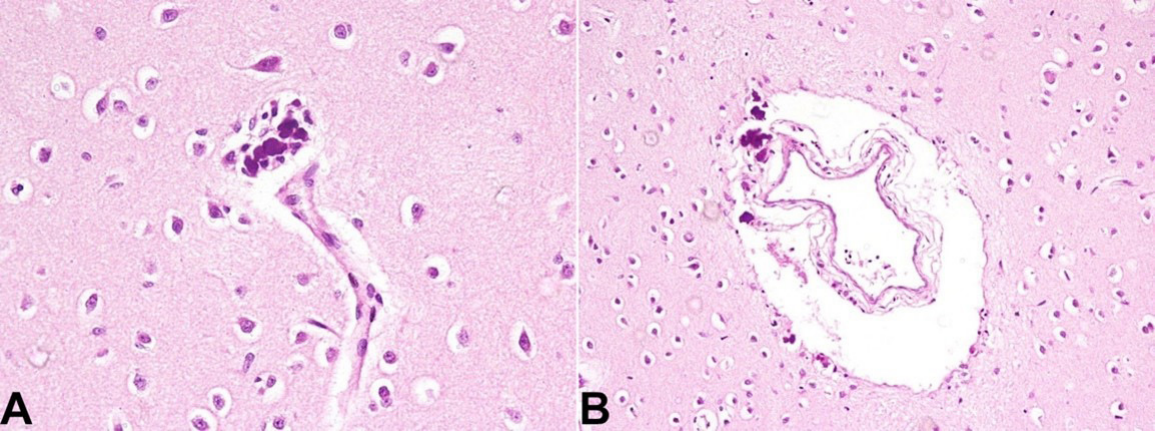
Photomicrograph of the cerebrum. **A** and **B** – Amphophilic calcific deposits seen around the blood vessels (H&E x400)

Calcification was also noted in the perivascular location of large as well as small blood vessels (Figures 3A and 3B). The perivascular calcification and presence of microglial nodules are features favoring CRS.

**Figure 4:**
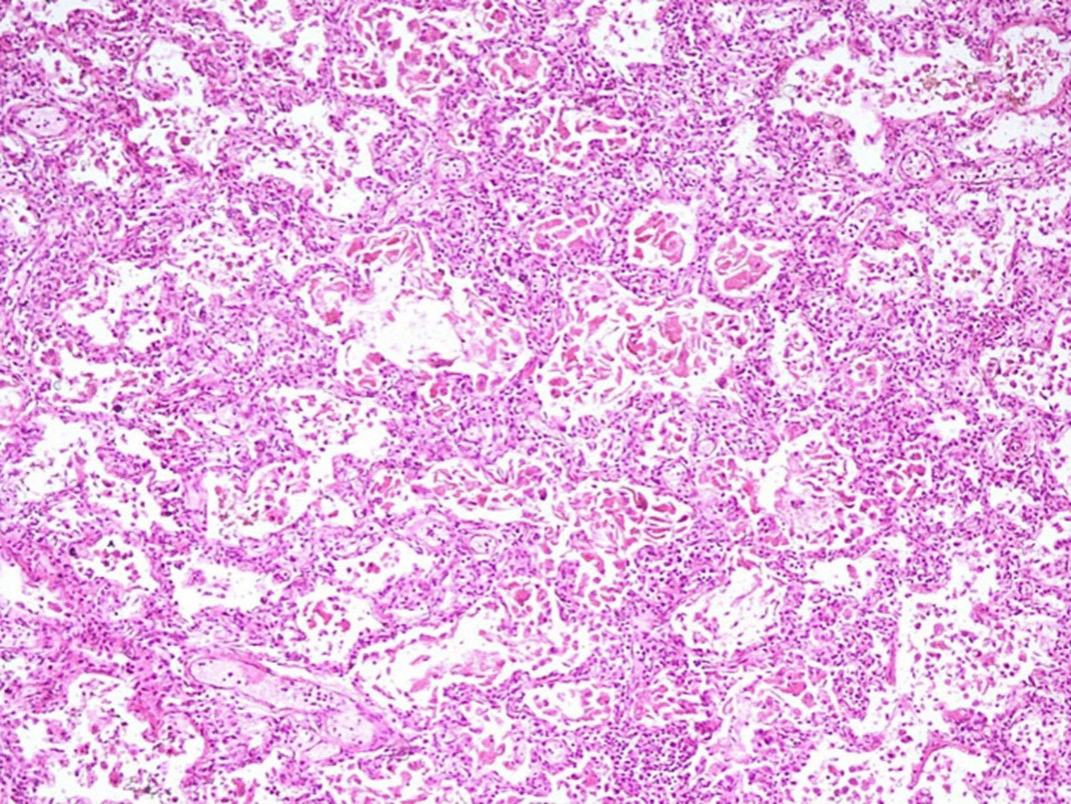
Photomicrographs of the lung. Low magnification showing interstitial widening and squamous epithelial cells and anucleated squames filling up the alveoli (H&E x40)

**Figure 5:**
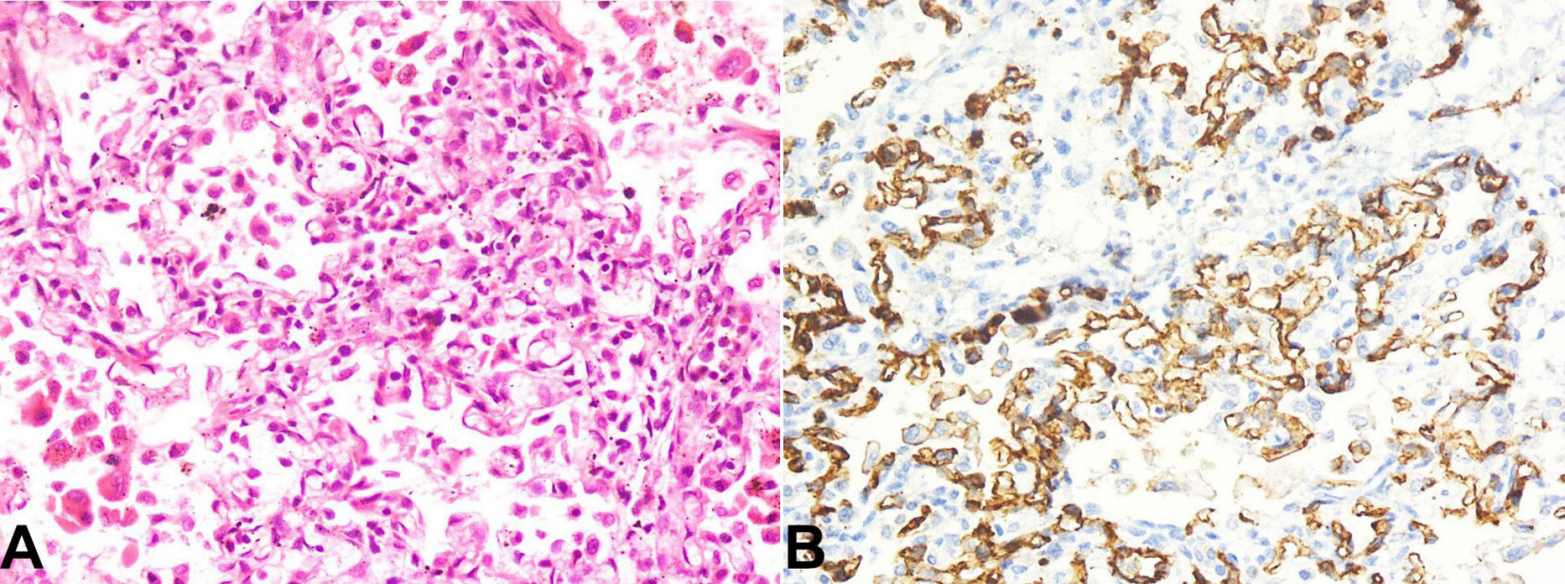
Photomicrographs of the lung. **A** – High magnification showing features of interstitial pneumonia with proliferating capillaries within the interstitium (H&E x400); **B** – CD31 immunostain highlights the abundance of capillaries within the interstitium (immunoperoxidase x400)

Both lungs were heavy and covered with dull pleura. On microscopy, they revealed alveolar spaces filled intra-alveolar squamous epithelial cells and anucleated squames, and focal presence of meconium pigment suggesting meconium aspiration (Figure 4). Further, interstitial pneumonia with interstitial widening due to moderate lymphomononuclear infiltrate was noted(Figures 5A) Focally multiple proliferating capillaries) were found (Figure 5B)

**Figure 6:**
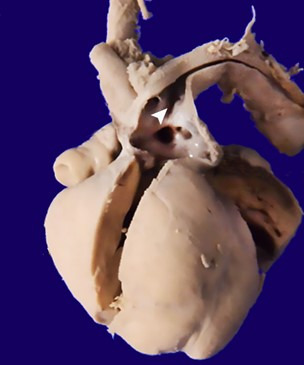
Gross view of the heart depicting the patent ductus arteriosus (arrowhead)

At some places, these proliferating capillaries were projecting into the alveolar spaces at places. The heart showed atrial septal defect (ASD) (ostium secundum type) of 3mm diameter and patent ductus arteriosus (Figure 6). All four chambers and the valves were grossly unremarkable. There was no evidence of myocarditis.

**Figure 7:**
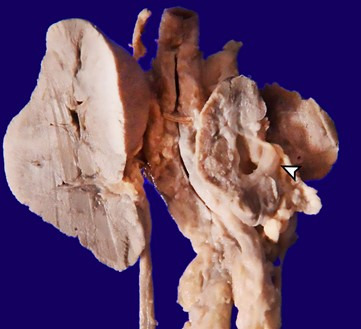
Gross view of the kidneys – note the right kidney depicting many tiny cysts within the cortex (arrowhead)

The right kidney weighed 4 g, and the left kidney weighed 10 g (Figure 7).

**Figure 8:**
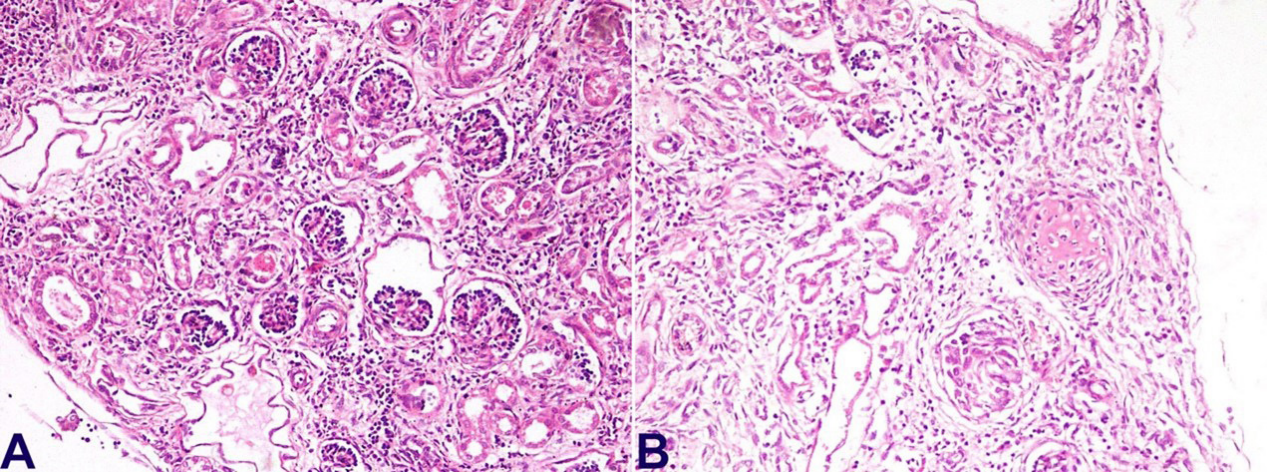
Photomicrographs of the kidney. **A** – Focal glomerulocystic change with dilated Bowman's space (H&E x200); **B** – Features of renal dysplasia with islands of cartilage noted in the right kidney (H&E x400)

The renal capsular surface showed fetal lobulations with a distinct cortico-medullary junction. In contrast, the cut surface of the right kidney showed dilated pelvis with pelvi-ureteric junction narrowing and pin-head-sized cysts both in the cortex and medulla (Figure 8A). The microscopy of the right kidney showed many cystic dilated tubules with patchy glomerulocystic change (Figure 8B).

The medulla showed many primitive ducts collared by immature mesenchyme and prominent medullary blood vessels. Occasional islands of cartilage were also noted in the cortex. The liver was grossly unremarkable with a patent biliary tract. However, the microscopy revealed a paucity of normal interlobular bile ducts with ductular proliferation at the periphery of portal tracts (interlobular bile duct: portal tract ratio 0.1). Only large portal tracts showed occasional normal interlobular bile ducts. Evidence of extramedullary hematopoiesis was also noted in the liver, along with small droplet macrovesicular steatosis patches. Other organs showed normal gross and microscopic morphology. Thus, the baby revealed pathological features consistent with congenital rubella syndrome, namely basal ganglia infarct, patent ductus arteriosus, atrial septal defect (ostium secundum type), and interstitial pneumonia. There was a paucity of interlobular bile ducts and evidence of renal dysplasia (right kidney) with pelvic-ureteric junction obstruction.

## DISCUSSION

Rubella infection is usually a mild and self-limiting illness in children and adults. However, in pregnant women, rubella infection during the first 20 weeks of pregnancy may result in severe fetal consequences such as miscarriage, stillbirths, intrauterine death, or a constellation of severe congenital birth defects, together called CRS. The risk of congenital infection and resultant birth defects is highest during the first 12 weeks of gestation, decreases after that, and is rare after 20 weeks of gestation.^1^ Rubella is an important public health problem. Worldwide, it is estimated that more than 100,000 babies are born with CRS each year.^2^ However, no nationwide data is available to know the actual burden of CRS in India. It is estimated that about 3-10% of suspected CRS cases are ultimately proven to have confirmed CRS. CRS accounts for 10-15% of pediatric cataracts, and 10-50% of children with congenital malformations have laboratory evidence of CRS.^3^ Serosurveys from various parts of India have found that 6-47% of schoolgirls aged 11-18 years and 12-30% of women in the reproductive age group are susceptible to rubella infection in India.^3^^,^ 4

CRS's common clinical features include cataract, congenital glaucoma, congenital heart disease, hearing impairment, pigmentary retinopathy, purpura, hepatosplenomegaly, microcephaly, and developmental delay. Infants with CRS often present with more than one sign or symptom consistent with congenital rubella infection.^5^ The case definition of CRS, approved by the Council of State and Territorial Epidemiologists and endorsed by the Centers for Disease Control and Prevention (CDC) for uniformity and epidemiological purpose, requires one of the symptoms mentioned above and laboratory evidence (isolation of rubella virus or detection rubella-specific IgM antibody). Even though no treatment exists for CRS, rubella is a vaccine-preventable disease. Rubella-containing vaccines (RCV) are highly immunogenic in adolescents and women. Therefore, to prevent CRS, ai is recommended to vaccinate all females in the reproductive age group with RCV.

In the index case, the sequential appearance of clinical findings delayed the diagnosis. The initial echocardiography showed tiny ductus arteriosus, which was attributed to prematurity. However, in the presence of persistent thrombocytopenia in an IUGR baby, the suspicion of structural PDA should have occurred and should have been evaluated for congenital rubella syndrome. To conclude, the persistence of unexplained thrombocytopenia in a small for gestational age infant should raise the suspicion of the TORCH group of infections and warrant detailed evaluation.
